# Reviews of Functional MRI: The Ethical Dimensions of Methodological Critique

**DOI:** 10.1371/journal.pone.0042836

**Published:** 2012-08-28

**Authors:** James Anderson, Ania Mizgalewicz, Judy Illes

**Affiliations:** National Core for Neuroethics, University of British Columbia, Vancouver, British Columbia, Canada; Mayo Clinic, United States of America

## Abstract

Neuroimaging studies involving human subjects raise a range of ethics issues. Many of these issues are heightened in the context of neuroimaging research involving persons with mental health disorders. There has been growing interest in these issues among legal scholars, philosophers, social scientists, and as well as neuroimagers over the last decade. Less clear, however, is the extent to which members of the neuroimaging community are engaged with these issues when they undertake their research and report results. In this study, we analyze the peer-reviewed review literature involving fMRI as applied to the study of mental health disorders. Our hypothesis is that, due to the critical orientation of reviews, and the vulnerability of mental health population, the penetrance of neuroethics will be higher in the review literature in this area than it is in the primary fMRI research literature more generally. We find that while authors of reviews do focus a great deal of attention on the methodological limitations of the studies they discussed, contrary to our hypothesis, they do not frame concerns in ethical terms despite their ethical significance. We argue that an ethics lens on such discussion would increase the knowledge-value of this scholarly work.

## Introduction

Neuroimaging studies involving human subjects, like other research involving human participants, raise a range of research ethics issues, including challenges related to informed consent [1], risk/benefit proportionality [2]–[4], and confidentiality [1]. Neuroimaging research also raises a number of neuroethical challenges more specific to imaging, such as complex issues related to incidental findings [4], and the use of general anaesthesia [5]. Moving beyond research ethics, neuroimaging raises a host of broader neuroethical challenges arising further down the translational pathway. These issues include the use of imaging in forensic and military contexts [6], commercialization for lie detection [7], clinical use [8], and – even more broadly – the impact of neuroimaging research on foundational concepts of selfhood [9], freedom and responsibility [10]–[12].

Many of these issues are heightened in the context of neuroimaging research involving persons with mental health disorders such as bipolar disorder and schizophrenia. Though persons suffering from these disorders may be competent to consent to research participation, competence cannot be presumed, complicating recruitment and consent procedures. Additional issues arise when neuroimaging studies are designed to image the brains of persons with florid symptoms, or persons suffering their first episode who have yet to receive treatment [13]. More generally, neuroimaging research involving persons with mental health disorders raises concerns about shaping or reinforcing essentialist conceptions of a pathological selfhood [14] with corresponding effects on the provision of care, social services, and legal status [15].

As the citations above suggest, there has been growing interest in these issues among legal scholars, philosophers, social scientists, and as well as neuroimagers over the last decade. Less clear, however, is the extent to which members of the neuroimaging community are engaged with these issues when they undertake their research and report results. Clarity on this question is crucial because only the neuroimaging community itself can create and sustain an ethical research culture. Research ethics committees (RECs) are “a resource, not the source, for ethical wisdom” [16] and neuroimagers themselves must take primary responsibility for the ethical aspects of their work.

To our knowledge, empirical, statistically defensible metrics of the extent to which neuroethical concerns have been taken up by the neuroimaging community had not yet been attempted prior to the work of our group. In a previous study, we analyzed the primary, peer-reviewed, functional magnetic resonance imaging (fMRI) research literature to determine the extent to which neuroimagers were engaged with ethical issues in their own research [17]. We hypothesized that the increasing popularity and media coverage of neuroimaging studies, and discourse surrounding real-world implications and early commercial applications, might have led to growing cross-citation between the fMRI and ethical, legal, and social issues (ELSI) literatures in recent years (1999–2009). Instead, we found very few citations of fMRI research by ELSI articles, and an even smaller number of fMRI articles that substantively cited the ELSI literature [17].

Though these findings were not entirely unexpected , they were concerning on a number of levels. First, as we have already emphasized, ethics review can supplement but not replace engagement by the neuroimaging community with ethical challenges raised by the conduct and results of imaging research [17]. Second, the absence of ethical discussion in the primary fMRI research literature obscures the care and concern researchers actually bring to ethical concerns when they conduct and report their research.

Here we examined the peer-reviewed review literature involving fMRI as applied to the study of mental health disorders. We hypothesized that, due to the critical orientation of reviews, and the vulnerability of mental health population, the penetrance of neuroethics would be higher in this literature than in the primary fMRI research literature more generally.

## Methods

We used an ISI Web of Science query to identify scholarly articles in the form of reviews, editorials, or related content found in academic journals, published between 1990 and 2010, and that refer to fMRI and one or more of “anxiety disorders,” “ADHD,” “mood disorders,” “personality disorders,” and “Schizophrenia or disorders with psychotic features”. The full query is provided in Appendix S1. The initial query identified 91 articles. This set was independently categorized by two of the investigators into three categories using the following key: ‘OF’ = Reviews of fMRI studies concerned with mental health; ‘USE’ = Reviews of mental health studies (of any kind) that use fMRI data; and ‘Excluded’ = Papers that are not (reviews AND involve fMRI AND concern mental health). The third investigator triple checked these categorizations and broke ties. At the end of this process, the set of 91 was categorized as follows: 51 ‘OF’ papers; 28 ‘USE’ papers; and 12 ‘EXCLUDED’ papers. 19 of the remaining 79 papers were not accessible at our institution and, for practical reasons, were also excluded, leaving 60 papers in total.

We adopted the coding scheme from our previous article [17] as a starting point, and added three new themes that emerged *in situ*: ‘therapeutic misconception,” “justice,” and “ethics” (to capture separately any explicit use of the word ‘ethics’?). Using these provisional codes, two of the authors double coded 10 randomly selected papers in order to validate the coding scheme. At the conclusion of this process, the authors augmented the coding scheme by exploding one of the original themes – Technical limitations, interpretation and validity of results – into three themes: methodological issues; medical complications; and technical or pragmatic considerations. All 60 papers were then double coded using the expanded coding scheme by two of three coders. Once all 60 papers were double coded, all three coders reviewed the results. Inconsistencies were identified and discussed until consensus was achieved.

**Figure 1:**
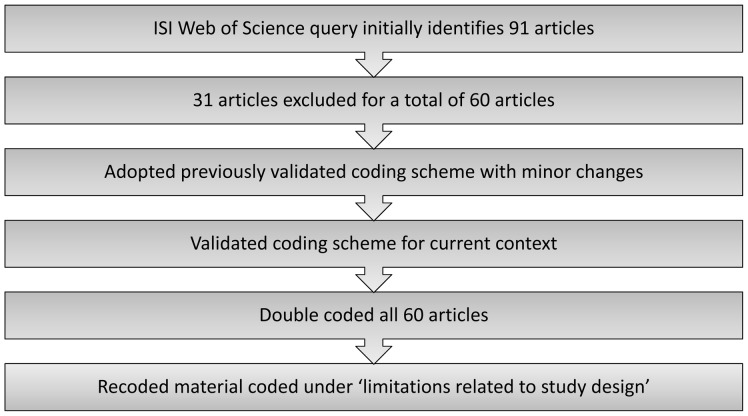
Methodological pathway – Illustration of the methodological steps for determining themes and codes for constant comparative analysis.

Results from the theme ‘methodological issues’ were then further subdivided into 5 additional sub-themes chosen because they featured prominently in the text coded under ‘methodological issues.’ The sub-themes are: limitations related to barriers to meta-analysis; limitations related to heterogeneous samples; limitations related to sample size; limitations related to variable reporting; and limitations related to cross sectional design. The text coded under ‘limitations to study design’ was then recoded using these five codes. Our methodological steps are summarized in Figure 1.

## Results

**Figure 2:**
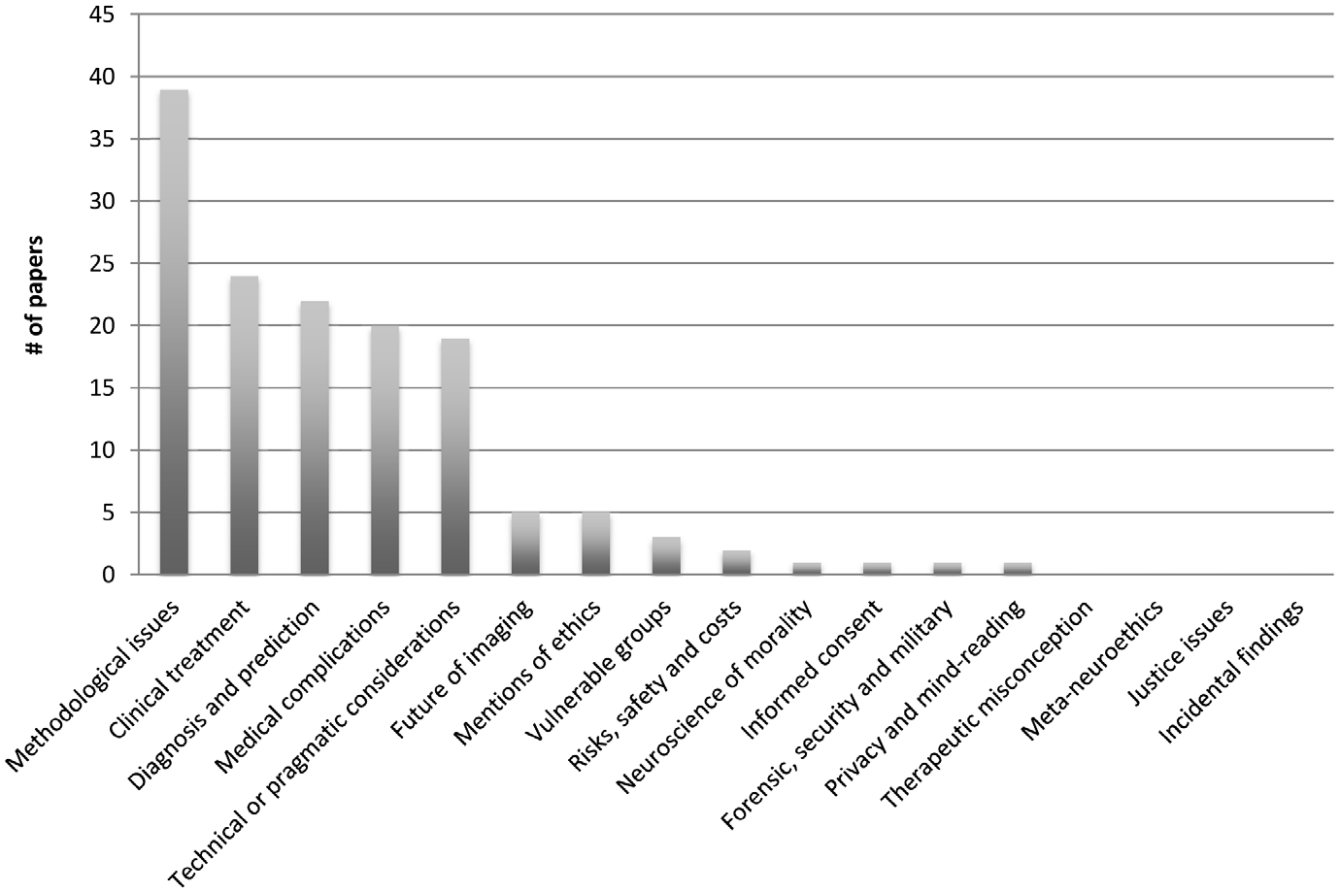
Distribution of coding themes – Illustration of the distribution of coding themes across the papers examined.

As illustrated in Figure 2, the review literature contained very little discussion of most of the themes included in our coding guide. Almost three quarters of the themes (71%) arose in five or less of the papers (≤8%) in our sample, and well over half (60%) of the themes arose in three or less (≤5%). Furthermore, almost one quarter of the themes (24%) arose either in one paper or in none at all.

Central ethical themes such as informed consent, risk, safety, and incidental findings were hardly mentioned by reviewers. The term ‘Informed consent’ arose in only one of the sixty review papers examined, ‘risk and safety’ arose in only two, and the issue of incidental findings – a central ethical concern in the context of fMRI research – was not mentioned in any of the reviews in our sample.

By contrast, a comparatively high number of papers mentioned ‘clinical treatment’ and ‘diagnosis and prediction’: 24 of 60 (40%) and 22 of 60 (36.7%) respectively. The theme most mentioned in the reviews, however, was ‘methodological issues.’ Thirty-nine of 60 papers (65%) discussed issues falling under this code. ‘Medical complications’ and ‘technical or pragmatic concerns’ also ranked in the top five codes mentioned, with 20 of 60 papers (33%) discussing the former theme, and 19 of 60 papers (31.7%) discussing the latter.

**Figure 3:**
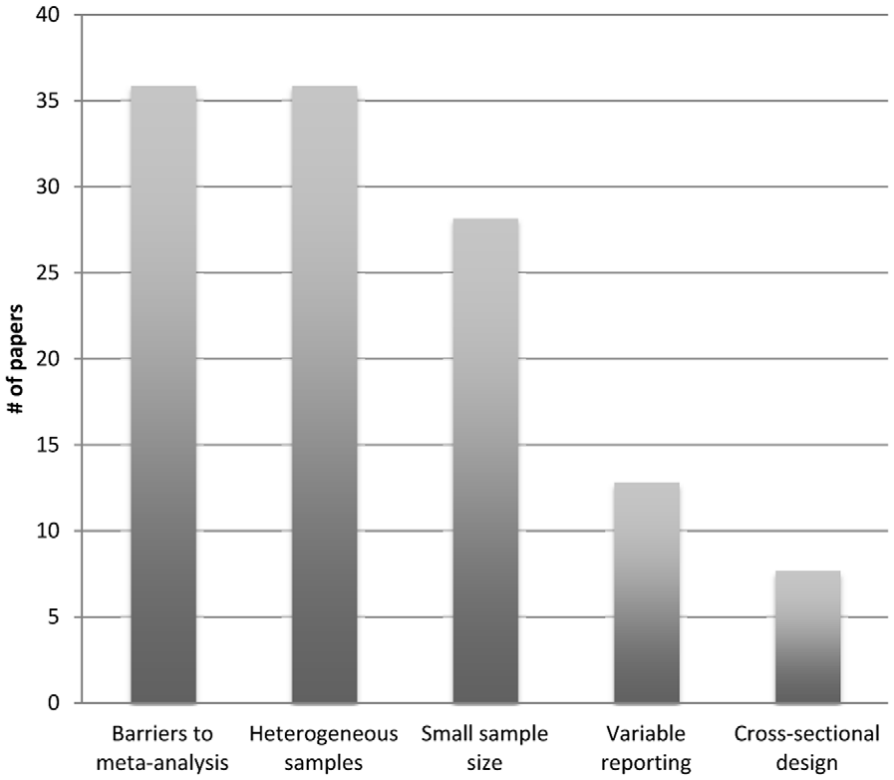
Distribution of subcodes – Illustration of the distribution of subcodes across the papers examined.

The results of our secondary analysis of the text coded under ‘methodological issues’ (Figure 3) yielded the following results: 14 of 39 papers (36%) mentioned ‘barriers to meta-analysis;’ 14 of 39 papers (36%) mentioned ‘heterogeneous samples;’ eleven of thirty-nine papers (28.2%) mentioned ‘small sample size;’ five of thirty-nine papers (12.8%) mentioned ‘variable reporting;’ and three of thirty-nine papers (7.7%) mentioned cross-sectional design’.

Specific quotes illustrate the three subthemes mentioned most prominently:

### Limitations related to barriers to meta-analysis

*“One possible explanation underlying discrepancies among the various studies lies in the actual design of the paradigms used to measure neural activity in patients with PTSD. According to our current research, 45 studies published used functional neuroimaging techniques in PTSD research. These studies make use of a wide range of methodologies: measuring resting brain activity, presenting a wide range of stimuli, and using active tasks performed by a subject. Within these main groupings there are further distinctions to be made, such as the type of stimulus (auditory, visual, trauma script, personal script), type of task (active recall, counting Stroop task, auditory continuous performance task), and type of tracer used in PET or SPECT studies. Due to this variation, difficulties arise when comparing data across different studies”* [18]

### Limitations related to heterogeneous samples

*“[A] significant number of studies included patients with bipolar type II disorder and bipolar disorder not-otherwise-specified (NOS). Mixing types of bipolar disorder is a significant confound as there is no a priori reason to assume that these… conditions share the same brain dysfunction. Finally, several studies do not describe the mood state of the bipolar patients during the MRI scan or they include samples that consist of patients in different mood states (i.e. manic, depressive, euthymic, and mixed states). This is a significant confound as fMRI studies have shown different brain activation patterns during different mood states… Overall, these potential confounds may explain some of the inconsistencies…”* [19].

### Limitations related to sample size

*“Finally, given the small samples size enrolled in the imaging studies here reviewed, the exact nature and extent of neuropsychological deficits observed in the high-risk states is not completely clear…”* [20].

*“[T]he power to detect neuropsychological and behavioural differences has often been limited by small samples size (generally less than 15 subjects per group)”* [21].

*“Because of the cost of neuroimaging research, there has been a tendency to publish underpowered studies, which effectively increases the prevalence of type I [sic] errors”* [22]

## Discussion

In order to measure the extent to which neuroimagers are engaged with ethical issues when they undertake their research and write up their results, we examined the peer-reviewed review literature involving fMRI as applied to the study of mental health disorders. We hypothesized that, due to the critical orientation of reviews, and the vulnerability of mental health population, the penetrance of neuroethics would be higher in the review literature in this area than it is in the primary fMRI research literature more generally.

Two major findings emerged from our analysis. First, contrary to our hypothesis, the penetrance of neuroethics in the review literature in this area is not significantly higher than it is in the primary fMRI research literature more generally. Reviewers rarely engage in explicit discussion of ethical issues associated with either the conduct or results of fMRI research on mental health disorders. Even central ethical themes, such as informed consent, risk, safety, and incidental findings are noted by reviewers only rarely.

The lack of discussion concerning core ethical themes with particular salience in fMRI research in this area is surprising. We expected to see some consideration of the challenges associated with working with vulnerable groups, as well as issues related to informed consent and risk. In actuality, these issues almost never arose in the reviews in our sample. Only 5% of the reviews we examined included discussion of the challenges associated with research involving vulnerable groups. Only two reviews included explicit discussion of the risks posed by the research under review. One review made reference to informed consent.

These findings are striking given that 42% of the reviews in our sample were about fMRI research on schizophrenia or disorders with psychotic features. Since researchers do not consistently exclude persons with florid psychosis from their studies, fMRI research involving this population is complicated by questions of competency. Furthermore, due to the severity of the symptoms associated with schizophrenia and other psychotic disorders, fMRI research may involve significant risks for participants, including the risks associated with treatment delays and/or washout periods [13].

Our second major finding is that, while reviewers rarely discuss ethical issues explicitly, they do discuss an important range of ethically salient issues in non-ethical terms. The best example of this trend is the widespread discussion of ‘methodological issues’ in the review literature. Sixty-five percent of papers discussed issues falling under this theme. Furthermore, as shown by our secondary analysis of the text coded at this node (i.e., methodological issues), the limitations mentioned were relatively consistent across the literature reviewed. Major sub-themes included limitations related to barriers to meta-analysis, limitations related to heterogeneous samples, and limitations related to sample size. Almost 50% of the papers in this subset raised concerns about barriers to meta-analysis; more than one-third highlighted concerns related to heterogeneous samples; and a third discussed limitations related to small sample sizes. All three themes were raised consistently across the ten-year span included in our sample.

Since reviews are generally supposed to offer critical assessment of the methodological quality of the literature reviewed, it is not particularly surprising that reviewers raise methodological concerns. The persistence of these concerns across the review literature is significant, however, and though reviewers did not couch their critical comments in ethical terms the issues raised are clearly of ethical importance.

Scientific quality is relevant from an ethical point of view because quality is tightly related to the potential knowledge-value of research and, thus, to the evaluation of risk-benefit proportionality. A study of low methodological quality is ethically problematic because it puts research subjects at risk and consumes scarce resources while producing results that are – at best – difficult to interpret.

The quality concerns noted by reviewers raise questions about the knowledge-value of the studies reviewed. A single scientific study is rarely sufficient to change minds or practice. Thus, the knowledge-value of a study is often a function of its amenability to combination with related work in the field. For this reason, barriers to meta-analysis – such as the heterogeneity of imaging equipment, imaging techniques, research methods, statistical approaches, baseline conditions, and reporting practices – constitute a significant threat to the knowledge-value of fMRI studies in this area.

Sample heterogeneity is also a significant concern. As reviewers frequently note, fMRI studies in this area often include patients with different clinical symptoms, disease subtypes, ages of onset, illness duration, severity of symptoms, medication status (including dosage and side effects), comorbid conditions, and substance abuse histories. Since it has been shown that these factors are related to brain functioning [23], [24], uncontrolled variation of these factors may well compromise the internal validity of studies. Sample heterogeneity, thus, constitutes a serious threat to the knowledge value of studies in this area.

Small sample sizes have long been a source of concern in fMRI research involving mental health disorders. Small samples diminish power and increase the probability of type II errors. Small sample sizes also prevent sub-group analysis, which might alleviate some of the concerns associated with heterogeneous samples. Finally, because of the significant barriers to meta-analysis found in this literature, combining studies cannot easily rectify this problem. Thus, small sample sizes also constitute a serious threat to the knowledge-value of this research.

Given the risks sometimes associated with participation in fMRI studies in this area [13], as well as the substantial resource commitments involved, these threats to knowledge-value are not just scientifically problematic – they are ethically problematic as well. Though reviewers highlighted the scientific import of these methodological concerns, as a group they did not comment on their ethical implications.

It is understandable, albeit unfortunate, that neuroscientists do not generally discuss the broader social, legal, and ethical implications of their work in the primary or review literature; the consideration of such issues, after all, is not central to their research. More worrisome, however, is the lack of discussion concerning ethical issues involved in the conduct of research itself. Ethical commitments shape the design and conduct of research directly via, e.g., recruitment procedures and risk minimization strategies, and studies may be poorly designed from an ethical point of view as well as a scientific point of view. Furthermore, the same problem may raise both scientific and ethical concerns. In today's research environment, ethical procedures and commitments are an integral part of the scientific process and should be treated as such.

In light of the severity, persistence, and consistency of the methodological concerns highlighted by the reviews of fMRI research in our sample, we believe the ethical import of these issues should be brought to light. Since the ethical implications of these issues are not addressed in the primary fMRI literature, authors of reviews would provide a valuable service to the neuroimaging community if they devoted some attention to these issues.

## Conclusion

In this study, we analyzed the peer-reviewed review literature involving fMRI as applied to the study of mental health disorders in order to determine the extent to which ethics concepts have penetrated neuroscience. We found that, contrary to our hypothesis, the penetrance of neuroethics in the review literature in this area is not significantly higher than it is in the primary fMRI research literature more generally. On the other hand, reviewers did focus a great deal of attention on the methodological limitations of the studies they reviewed. They did not, however, frame these concerns in ethical terms despite their ethical significance. More explicit discussion of the ethical implications of the methodological concerns would increase the knowledge- value of these studies significantly.

## Supporting Information

Appendix S1 (DOCX)
