# Supplementary material for: Reviews of Functional MRI: The Ethical Dimensions of Methodological Critique
Source: PLoS One. 2012 Aug 28;7(8):e42836. doi: 10.1371/journal.pone.0042836 (PMC3429464; doi:10.1371/journal.pone.0042836)
Supplement: Appendix S1 — (DOCX) [file pone.0042836.s001.docx]

Appendix 1: Search Query

Full query: (("magnetic resonance imaging"[mesh] AND Journal Article[ptyp] AND ("fmri"[Title/Abstract] OR "Functional MRI"[Title/Abstract] OR "Functional magnetic resonance imaging"[Title/Abstract] OR "Functional MR Imaging"[Title/Abstract])) AND (Editorial[ptyp] OR Letter[ptyp] OR "Practice Guideline"[ptyp] OR Review[ptyp] OR Comment[ptyp]) AND ("1990"[PDAT] : "2010"[PDAT]) AND English[lang]) AND ("Anxiety Disorders"[mesh] OR "Attention Deficit Disorder with Hyperactivity"[mesh] OR "Mood Disorders"[mesh] OR "Personality Disorders"[mesh] OR "Schizophrenia and Disorders with Psychotic Features"[mesh])
